# Comparison of two fluorescent probes in preclinical non-invasive imaging and image-guided debridement surgery of Staphylococcal biofilm implant infections

**DOI:** 10.1038/s41598-020-78362-7

**Published:** 2021-01-15

**Authors:** Howard Y. Park, Stephen D. Zoller, Vishal Hegde, William Sheppard, Zachary Burke, Gideon Blumstein, Christopher Hamad, Marina Sprague, John Hoang, Ryan Smith, Francisco Romero Pastrana, Julie Czupryna, Lloyd S. Miller, Marina López-Álvarez, Mafalda Bispo, Marleen van Oosten, Jan Maarten van Dijl, Kevin P. Francis, Nicholas M. Bernthal

**Affiliations:** 1 Department of Orthopaedic Surgery, David Geffen School of Medicine at University of California Los Angeles, 1250 16th Street, Suite 2100, Santa Monica, CA 90404 USA; 2 PerkinElmer, 68 Elm Street, Hopkinton, MA 01748 USA; 3 Department of Medical Microbiology, University of Groningen, University Medical Center Groningen, Hanzeplein 1, Groningen, 9700 RB The Netherlands; 4 Department of Dermatology, Johns Hopkins University School of Medicine, Baltimore, MD USA; 5 Department of Orthopaedic Surgery, Johns Hopkins University School of Medicine, Baltimore, MD USA

**Keywords:** Biofilms, Infectious diseases

## Abstract

Implant-associated infections are challenging to diagnose and treat. Fluorescent probes have been heralded as a technologic advancement that can improve our ability to non-invasively identify infecting organisms, as well as guide the inexact procedure of surgical debridement. This study’s purpose was to compare two fluorescent probes for their ability to localize *Staphylococcus aureus* biofilm infections on spinal implants utilizing noninvasive optical imaging, then assessing the broader applicability of the more successful probe in other infection animal models. This was followed by real-time, fluorescence image-guided surgery to facilitate debridement of infected tissue. The two probe candidates, a labelled antibiotic that targets peptidoglycan (Vanco-800CW), and the other, a labelled antibody targeting the immunodominant *Staphylococcal* antigen A (1D9-680), were injected into mice with spine implant infections. Mice were then imaged noninvasively with near infrared fluorescent imaging at wavelengths corresponding to the two probe candidates. Both probes localized to the infection, with the 1D9-680 probe showing greater fidelity over time. The 1D9-680 probe was then tested in mouse models of shoulder implant and allograft infection, demonstrating its broader applicability. Finally, an image-guided surgery system which superimposes fluorescent signals over analog, real-time, tissue images was employed to facilitate debridement of fluorescent-labelled bacteria.

## Introduction

Orthopaedic implant infections are clinically disastrous for patients and economically burdensome for the health system^1–4^. Once bacteria adhere to avascular implants and form protective biofilms, they become highly recalcitrant to systemic and local antibiotics. Thus, biofilm-associated implant infections across all medical and surgical disciplines often require a combination of debridement surgery of wound beds, explantation of implants, and long-term antibiotic treatment^5–7^. Despite these aggressive surgical interventions, treatment failure is common and highly morbid to the patient^5–7^. The current approach to debride infected tissue is imprecise and rudimentary at best, consisting of excision of visually pathologic tissue at the discretion of the surgeon. Therefore, due to the inability to discriminate infected from native tissue, reoperations with serial debridements are commonplace, escalating costs and risks to the patient^8–10^.

Accurate diagnosis and treatment of biofilm infections remains a challenge due to difficulties with localization and identification of bacterial pathogens seated deep within tissues. Many imaging modalities including X-ray (XR), ultrasound (US), computer tomography (CT), or magnetic resonance imaging (MRI) are limited in their ability to achieve high resolution adjacent to an implant; whereas nuclear medicine methodologies, like positron emission tomography (PET), cannot accurately discriminate infection from other entities, such as sterile inflammation or fluid collections^11–15,39^. Furthermore, the current standard of infection diagnosis by culture growth of the pathogen suffers from false-negative and false-positive culture results at alarmingly high rates, and there exist inherent risks to the patient with invasive procurement of culture samples^13,15–17^. As a result, current treatment algorithms of implant infections are often based on clinical judgments that utilize diagnostics that are known to be unreliable.

The development of fluorescent molecular targeting probes that specifically bind to specific bacterial pathogens has been widely heralded as a major advancement to improve diagnosis and treatment of biofilm infections^18^. *Staphylococcus* species are identified as the most common pathogens of bacterial implant infections^19–21^. Vancomycin-IRDye800CW (Vanco-800CW) consists of fluorescently-labelled vancomycin, an antibiotic that binds to peptidoglycan in the cell wall of Gram-positive bacteria^22^. In contrast, the immunodominant staphylococcal antigen A (IsaA) antibody labelled with a cyanine 680 dye (1D9-680) is a fluorescent probe that labels ubiquitously expressed proteins involved in cell wall metabolism of *S. aureus*, but not other types of bacteria^23^. In addition, IsaA is bound by the IgG-binding proteins Spa and Sbi of *S. aureus*^23^. Both molecular targeting probes have been shown to accurately label *S. aureus* bacteria within in vitro and in vivo models^22,23^. Targeted fluorescent imaging (TFLI) applied to these novel staphylococcal targeting molecules can, thus, illuminate infections in vivo with the potential to increase diagnostic sensitivity and specificity. Furthermore, image-guided surgical debridement, achieved using a specialized fluorescence camera system in the surgical suite, may improve the accuracy of infection excision, which currently relies solely on the surgeon’s discretion based on imprecise visual inspection of infected tissue.

The purpose of this study is to: (1) compare the diagnostic accuracy of two candidate probes targeting *Staphylococcus aureus*, (2) assess the broader applicability of the more specific probe in the spinal implant model to identify *S. aureus* infections in other models of implant-associated infection, and (3) test the functionality of the probe within a real-time, fluorescence image-guided surgery system in a proof-of-concept study evaluating the feasibility of image-guided infection debridement surgery.

## Results

### In vitro assessment of staphylococcal biofilm targeting using Vancomycin- and 1D9 antibody-based fluorescent probes

**Figure 1:**
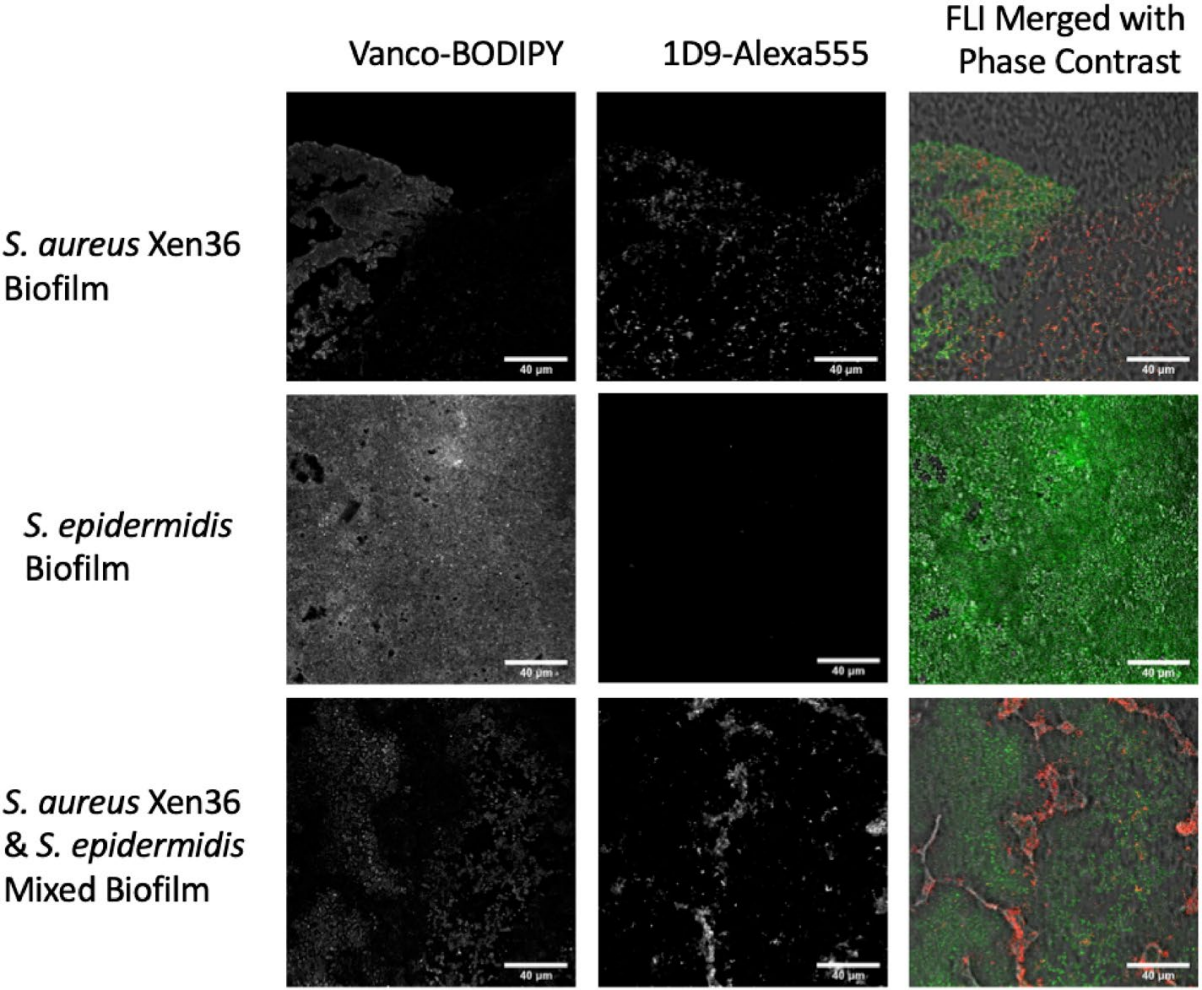
Imaging of staphylococcal biofilms with vancomycin- and 1D9-based fluorescent probes. Biofilms of *S. aureus* Xen36*, S. epidermidis* ATCC 38984*,* or a mixture of both *Staphylococcus* species were grown on cover slides for confocal microscopy. The established biofilms were simultaneously incubated with the Vanco-BODIPY and 1D9-Alexa555 probes. Subsequently, images were recorded by confocal fluorescence microscopy, revealing binding of the vancomycin probe to all biofilms and the 1D9-Alexa555 probe to *S. aureus* biofilms only. Of note, we attribute the “patchy” fluorescence pattern of the S. aureus cells in the mixed biofilm, as detected with the 1D9-Alexa555 probe, to the fact that S. epidermidis secretes serine proteases that can inhibit biofilm formation by S. aureus^41^. The magnification is indicated by scale bars.

Staphylococcal biofilms composed of either *S. aureus*, *S. epidermidis* or a mixed culture of both strains were established on the surface of 18 mm chemically resistant borosilicate glass coverslips. These biofilms were then incubated with vancomycin-BODIPY FL (Vanco-BODIPY) and 1D9-AlexaFluor555 (1D9-Alexa555) simultaneously*.* Of note, the latter two probes were implemented for the in vitro assessment of staphylococcal biofilm targeting, because they are more suitable for the fluorescence microscopic analysis of biofilms than the equivalent Vanco-800CW and 1D9-680 probes used for subsequent in vivo imaging of infection. As shown in Fig. 1, the vancomycin probe is capable of binding to both *S. aureus* and *S. epidermidis*. In contrast, the 1D9 probe binds exclusively to *S. aureus,* because this strain exposes the IsaA epitope on its surface, whereas the selected *S. epidermidis* strain is incapable of IsaA expression. Interestingly, Vanco-BODIPY appears to be more density/biomass-dependent, with the probe seeming to penetrate deeper into the biofilm matrix compared to 1D9, which appears more surface-bound (top panels of Fig. 1). This may relate to the fact that the expression of IsaA is downregulated in staphylococal biofilms and so 1D9 may bind preferentially to more actively growing bacteria on the surface of the biofilm^38^. The apparently lower biofilm penetration by the 1D9 probe compared to the vancomycin probe may also relate to the fact that the full-size 1D9 IgG1 molecule is substantially larger in size than vancomycin.

### Establishing infected spinal implants within a mouse model

**Figure 2:**
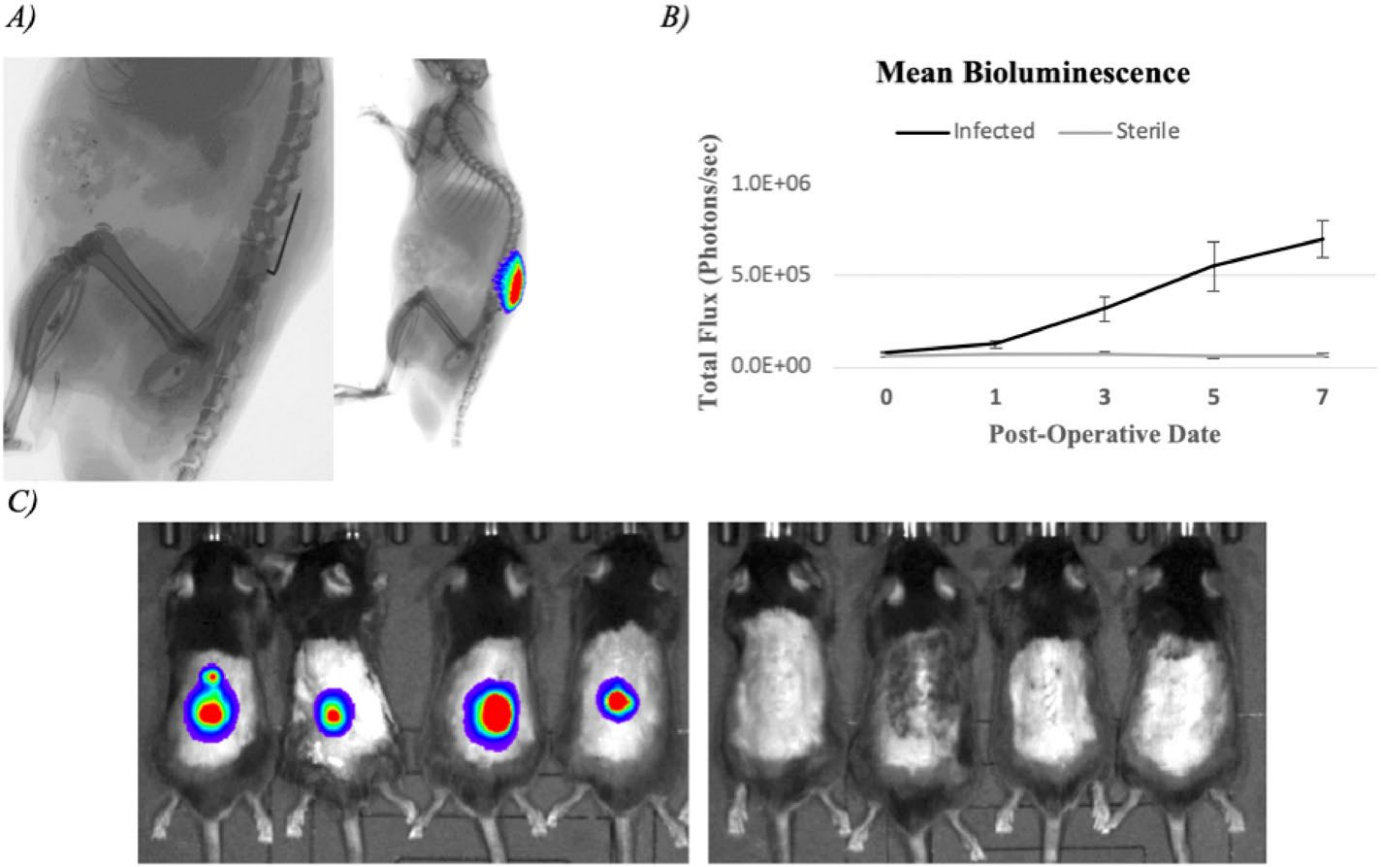
Spinal implant infection model. (**A**) Post-operative day 7 (POD 7) lateral XR revealing the position of the pin fixed within the spinous process in the lumbar spine and overlying bioluminescent signal of *S. aureus* Xen36. (**B**) Mean total bioluminescence represented as total flux (photons/sec) at each POD with error bars derived from Standard Error of the Mean for the Infected (n = 10) and Sterile (n = 5) cohorts. (**C**) Representative bioluminescence images of four mice from the Infected and Sterile Cohorts at post-operative day 7.

To compare the abilities of the Vanco-800CW and 1D9-680 probes in diagnosing implant infections, an established mouse model of spine implant biofilm infection was employed^24^. Briefly, in this infection model a midline skin incision measuring ~ 2 cm centered over the lumbar spine was made followed by exposure of the spinous processes along the entire length of the incision. Once the spinous processes were exposed, an L-shaped stainless-steel implant was fixed into the spinous process and inoculated with a bioluminescent *S. aureus* (Xen36 strain) (Fig. 2A)*.* 10 mice were subjected to the surgery as described and 5 mice were subjected to this surgery without inoculation of *S. aureus,* receiving an equivalent volume of sterile saline. Non-invasive in vivo bioluminescence imaging (BLI) was performed on post-operative days (POD) 0, 1, 3, 5, and 7 to assess the bacterial burden, which showed that the mean in vivo BLI signal of the Xen36 bacteria was significantly higher in infected mice compared to sterile mice at all POD time-points (*P* < 0.05) (Fig. 2B). All 10 mice in the infection group were verified to have spinal infections and all 5 control mice had no evidence of infection, as determined by in vivo BLI signal detection on POD 0 (Fig. 2C).

### Candidate probes administered to mice and subsequent two-dimensional imaging results

**Figure 3:**
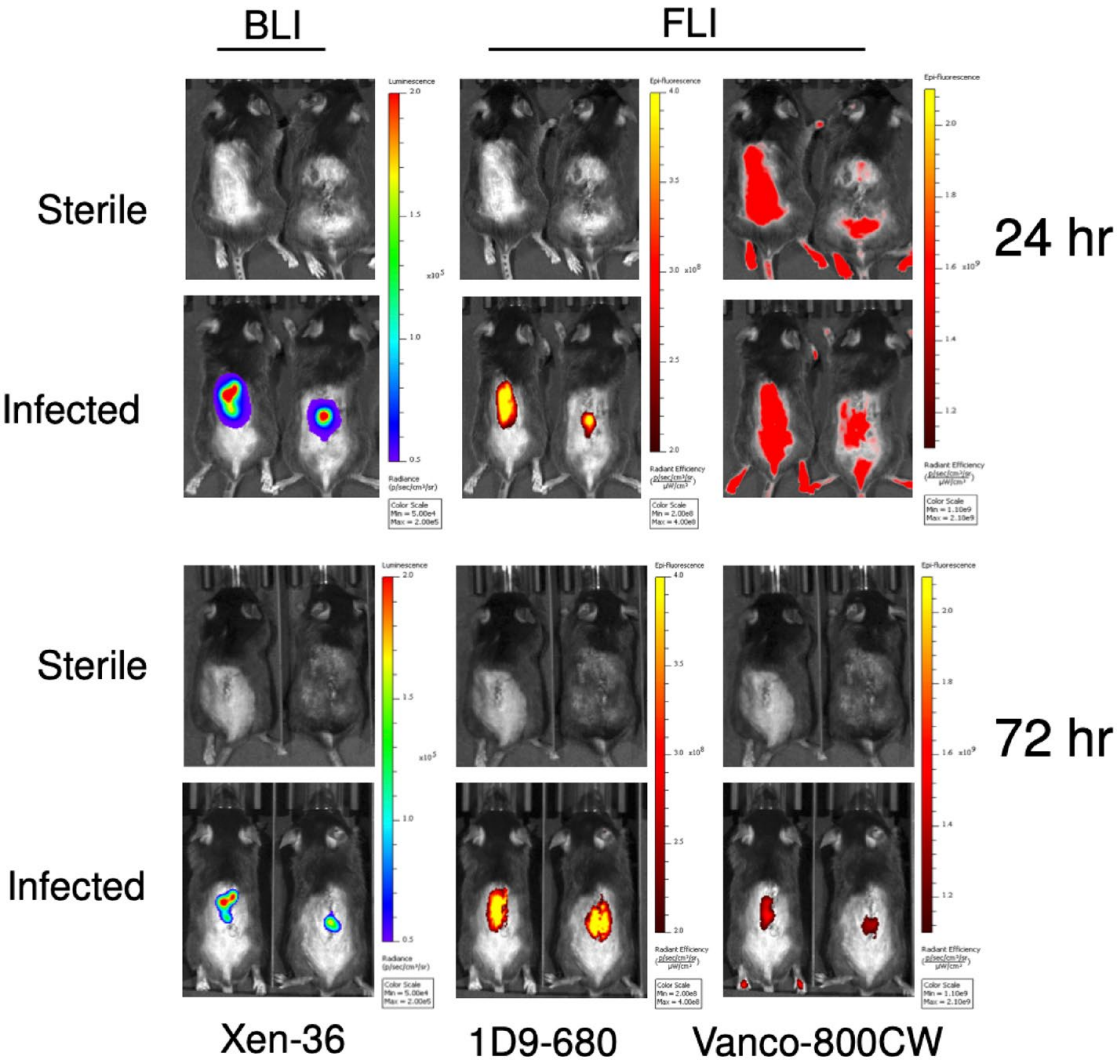
Bioluminescence and fluorescence images of mice with spinal implant infection at 24 h and 72 h post probe injection (PI). Bioluminescence imaging of two representative mice reveals bacterial signal about the lumbar spine. At 24 h PI, the 1D9-680 probe was imaged with an excitation/emission pair of 675/720 nm revealing a similar pattern of signal in comparison to bioluminescence. The Vanco-800CW probe was imaged with an excitation/emission pair of 745/800 nm revealing a relatively non-specific signal about the lumbar spine as well as caudally to the base of the tail and feet due to the ubiquitous pooling of the probe within the tissues as a consequence of the pharmacokinetics of the Vanco-800CW probe. Clearance of this pooling effect was resolved by 72 h PI with remaining probe signal localizing to the site of infection. By 72 h PI, the 1D9-680 and Vanco-800CW signals both revealed a similar pattern in comparison to bioluminescence.

On POD 7, which was previously shown to correlate with the peak of infection in our prior work, the 10 infected and 5 sterile control mice were subjected to injections of both the Vanco-800CW and 1D9-680 fluorescently labelled probes^18,23^. Mice were imaged for both in vivo BLI and in vivo fluorescent imaging (FLI) signals, 24-, 48- and 72-h post-injection (PI) of the probes. In vivo FLI was performed at excitation/emission wavelengths of 675/720 nm and 745/800 nm, corresponding to the 1D9-680 and Vanco-800CW fluorescent probes, respectively. Importantly, these probes did not exhibit spectral overlap; thus upon co-injection in vivo, an accurate, head-to-head comparison could be made of their accuracy and specificity of targeting the *S. aureus* infection. At 24 h PI of the probes, in-vivo FLI revealed a highly intense signal from both probes (Fig. 3). However, in the case of the Vanco-800CW probe, this signal still lacked resolution and was found to be equally as strong in the sterile control animals due to non-specific tissue distribution. By 72 h PI of the probes, the Vanco-800CW signal appeared to be accumulating at the site of the bacterial infection to a similar degree as the 1D9-680 probe when the animals were imaged two-dimensionally (2D) in the dorsal plane. In contrast to the apparent slower clearance of the Vanco-800CW probe, the 1D9-680 probe appeared to specifically target the *S. aureus* infections as early as 24 h PI. Moreover, the antibody probe seemed to clear more rapidly from the animal’s tissues, since none of the sterile control mice showed non-specific background signals, as seen with the Vanco-800CW probe (Fig. 3). Possibly, this relates to the fact that 1D9-680 is cleared by the liver, while the Vanco-800CW probe is excreted via the kidney and bladder^22,23^.

### Three-dimensional computed tomography scans with bioluminescent and fluorescent overlay

**Figure 4:**
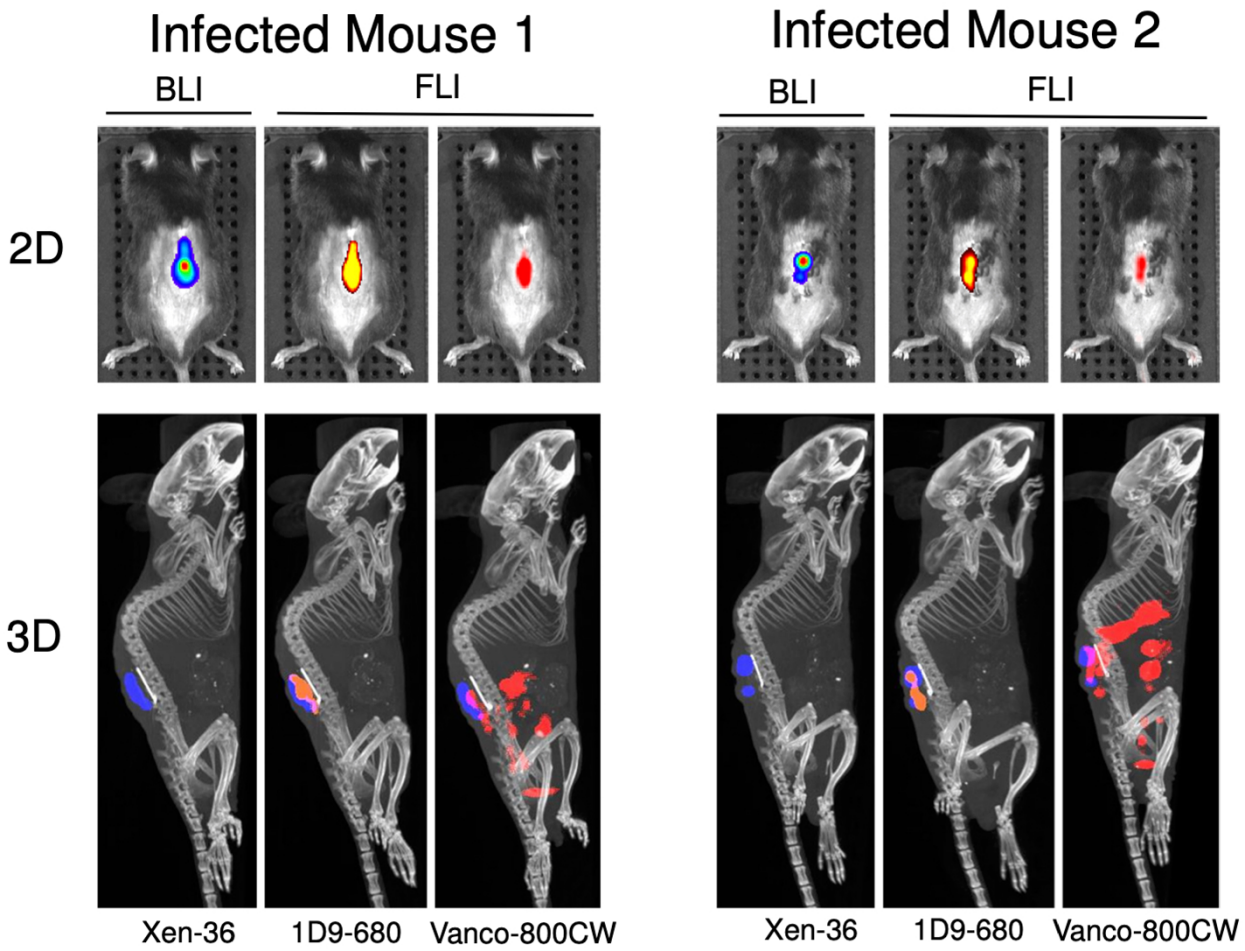
Three-dimensional bioluminescence and fluorescence imaging of spinal implant infection. At 72 h following PI, the mice were subjected to 3D-CT with bioluminescence and fluorescence imaging. Two representative infected mice are shown. The 1D9-680 probe signal colocalizes with the bioluminescent bacterial signal on the infected implant. The Vanco-800CW probe signal colocalizes with the infected implant, but is also detected in the pelvic and abdominal cavities.

In order to assess the distribution of the 1D9-680 and Vanco-800CW probes throughout the mouse, three-dimensional (3D) computed tomography scans with 3D in vivo BLI and FLI overlay were completed at 24-, 48- and 72-h PI of the probes. The 1D9-680 probe’s fluorescence signal tightly correlated with the in vivo BLI signals in all planes from 48 h PI onwards (Fig. 4). In contrast, the Vanco-800CW probe’s fluorescence signal was less discriminatory. Although it clearly accumulated at the site of the bacterial in vivo BLI signals, it was also detectable in the abdomen and pelvis of the animals. This off-target accumulation of Vanco-800CW in the mice is possibly due to non-specific retention in tissues or the bladder, non-specific targeting by breakdown products of the probe, and/or dissemination of bacterial debris towards the murine abdomen. Importantly, this 3D assessment revealed better performance by 1D9-680 in terms of accuracy and specificity of co-localization with living bacteria within the biofilm on spinal implants.

### Ex vivo confirmation

**Figure 5:**
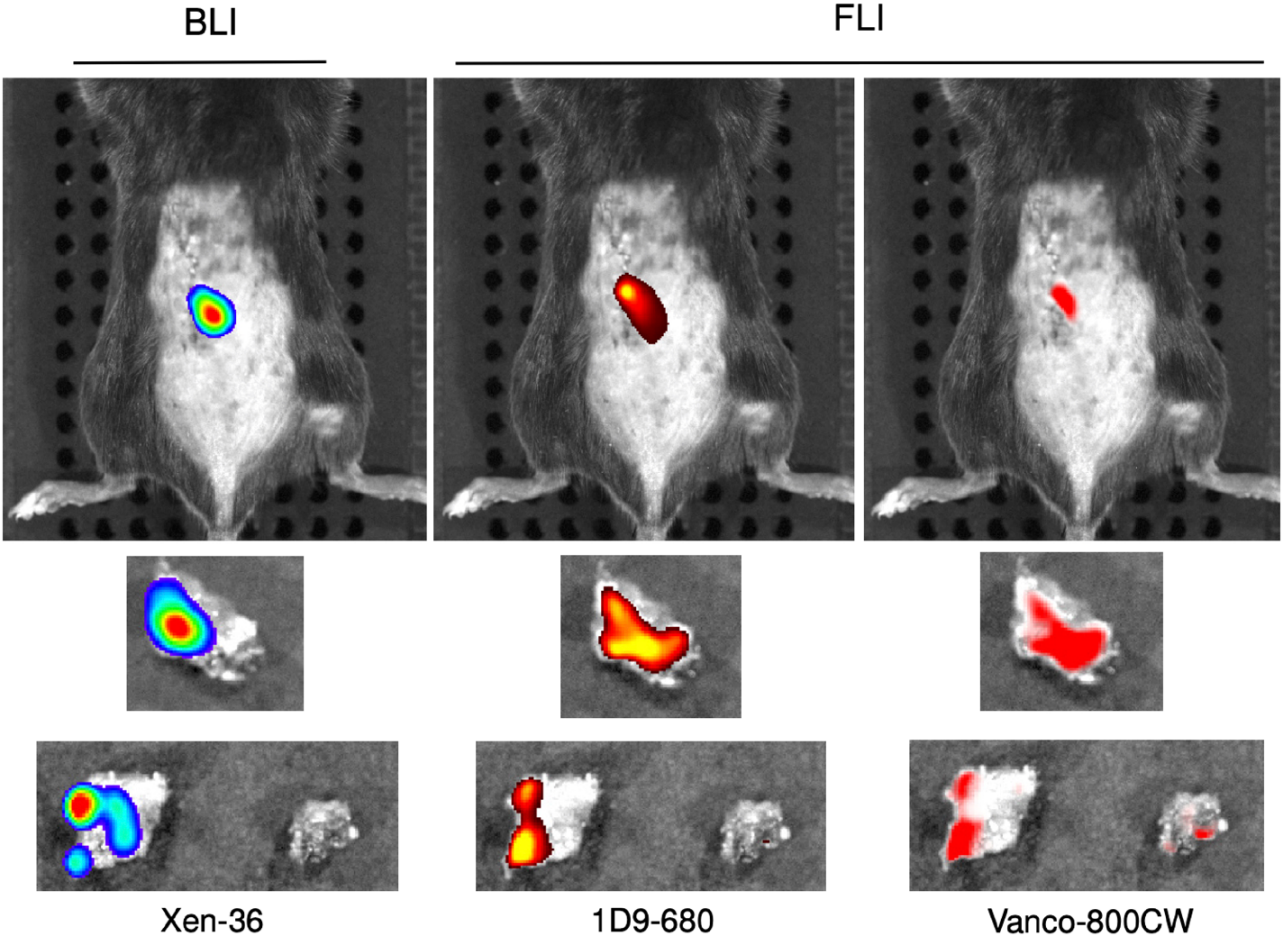
Bioluminescence and fluorescence imaging of infected necropsy specimen from spinal implant infection. Following necropsy of the infected tissue bed, bioluminescence and fluorescence imaging were repeated revealing co-localization of the 1D9-680 probe with infected tissue. As shown for a representative mouse, the Vanco-800CW imaging revealed co-localization with the infected necropsy specimen, but fluorescence signal was also detected on supplemental tissue without bioluminescence.

At 72 h PI of the probes, following 3D imaging, necropsy surgery was performed to determine the ex vivo co-localization of the probes with the normative ex vivo BLI signals. Both 1D9-680 and Vanco-800CW colocalized with the ex vivo BLI signals of the bacteria in the explanted periprosthetic bone (Fig. 5, Middle Row), however, the Vanco-800CW also localized to an uninfected piece of bone that showed no presence of living bacteria by ex vivo BLI (Fig. 5, Bottom Row). This again shows the adequate sensitivity of both probes, but demonstrates a higher specificity of the 1D9-680 probe for living bacteria in the present infection model.

### Assessing broader applicability of the 1D9-680 probe

**Figure 6:**
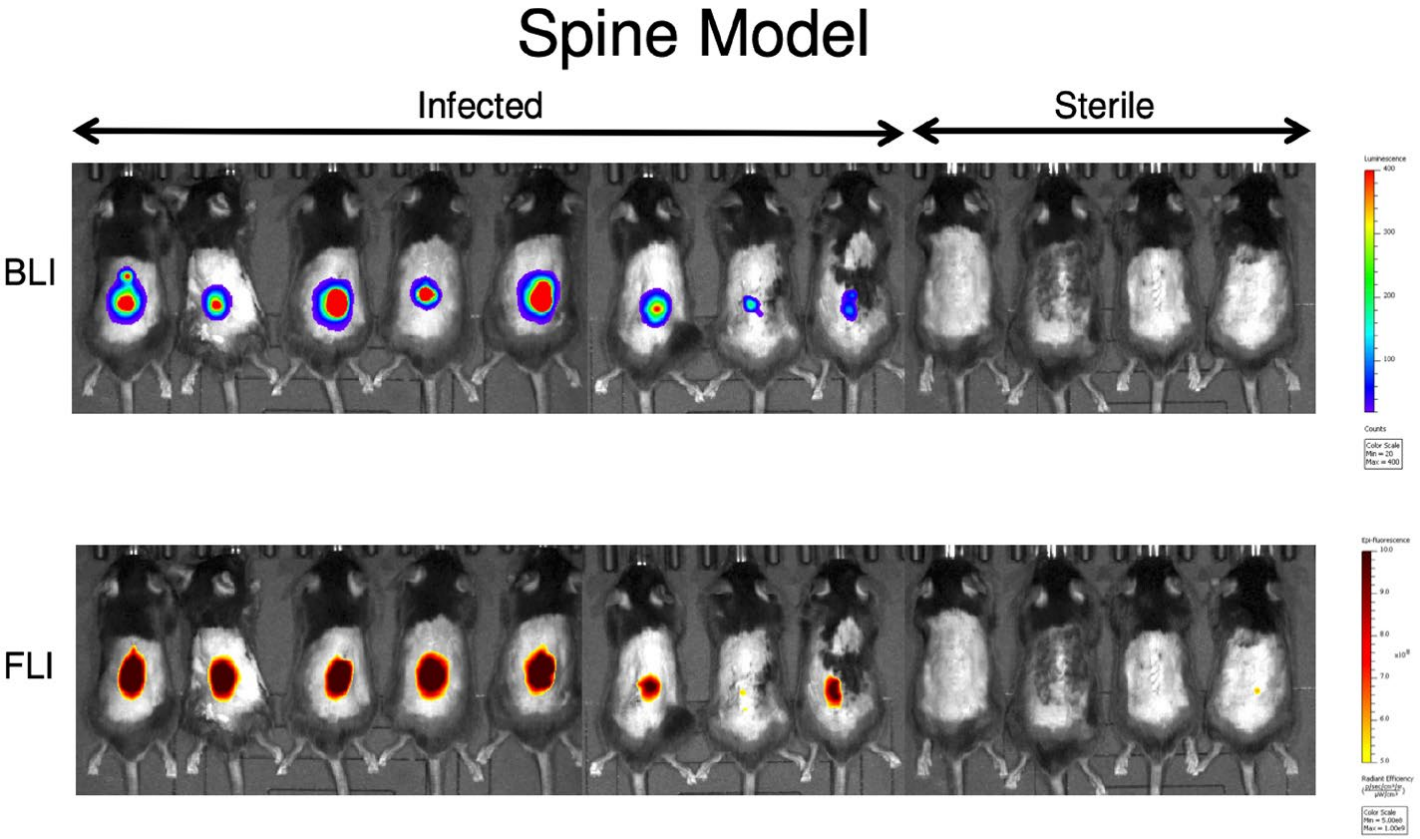
Bioluminescence and fluorescence imaging of mice with a spinal implant infection 72 h post 1D9-680 probe injection. At 72 h PI of the 1D9-680 probe, co-localization of the probe in 8 mice with infected implants is evidenced by bioluminescence and fluorescence imaging as described for Fig. 3. As expected, neither bioluminescence nor fluorescence are detectable in the 4 control mice with sterile spinal implants.

Given the off-target binding of Vanco-800CW demonstrated in Fig. 3 and the greater specificity of 1D9-680 demonstrated in Fig. 4 (Bottom row), all additional experiments involved assessing only the 1D9-680 probe. Since all prior spinal implant infection imaging studies had been conducted using both 1D9-680 and Vanco-800 in combination, a second group of 8 infected mice and 4 control mice were intravenously injected with the 1D9-680 probe alone, to allow for an independent assessment of this antibody probe (Fig. 6). Based on previous imaging results, all 12 mice were imaged 48 h PI. In Fig. 6, 1D9-680 co-localized consistently with the Xen36 in vivo BLI signals. Moreover, the magnitude of the in vivo FLI signals were somewhat comparable to the in vivo BLI signals, with only the weakest in vivo BLI signals showing in vivo FLI signals close to background levels (mouse #7 from the left) and all sterile control mice demonstrated negligible background in vivo FLI signals.

**Figure 7:**
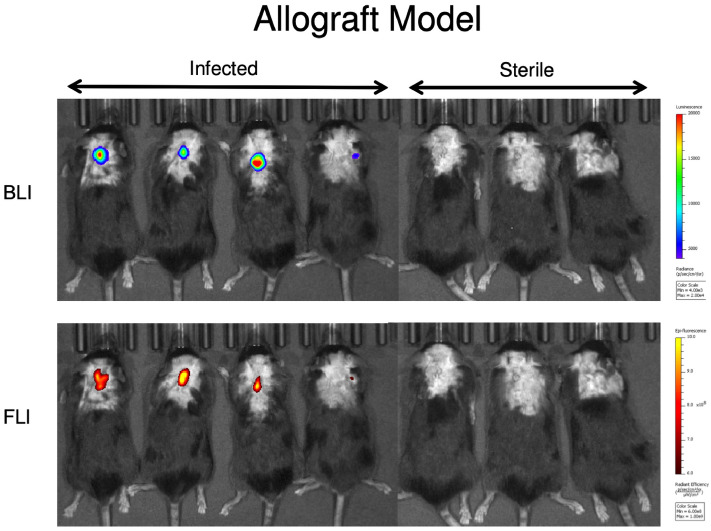
Bioluminescence and fluorescence imaging of mice with a spinal allograft infection 72 h post 1D9-680 probe injection. At 72 h PI of the 1D9-680 probe, co-localization of the probe in 4 mice with infected dorsally placed cancellous human allograft implants is evidenced by bioluminescence and fluorescence imaging as described for Fig. 3. As expected, neither bioluminescence nor fluorescence are detectable in the 3 control mice with sterile allograft implants.

**Figure 8:**
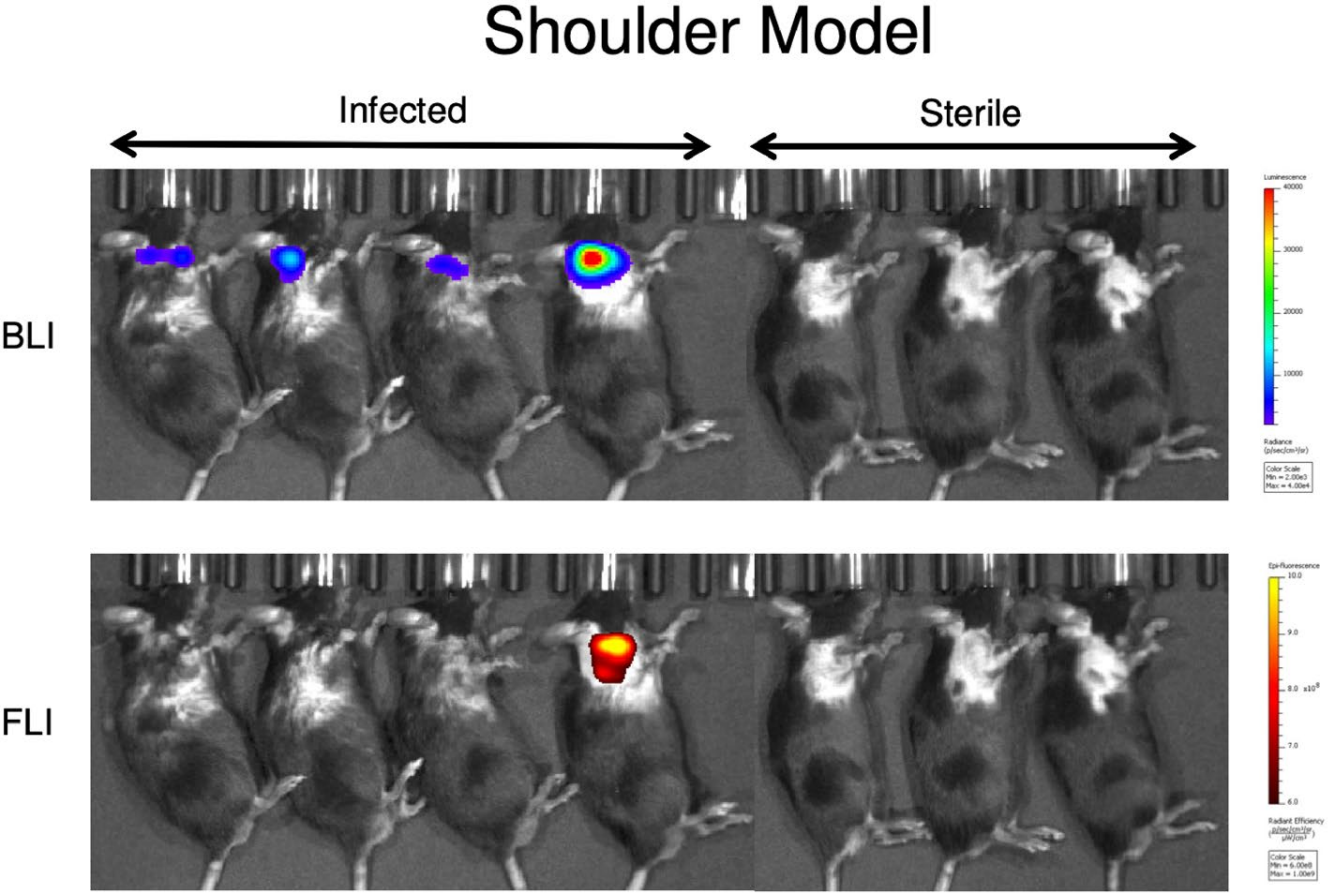
Bioluminescence and fluorescence imaging of mice with a spinal allograft infection 72 h post 1D9-680 probe injection. At 72 h PI of the 1D9-680 probe, co-localization of the probe in 1 out of 4 mice with infected shoulder arthroplasty is evidenced by bioluminescence and fluorescence imaging as described for Fig. 3. As expected, neither bioluminescence nor fluorescence are detectable in the 3 control mice with sterile shoulder arthroplasty.

Next, the 1D9 probe was tested in two additional established models of implant-associated infection: an allograft (Fig. 7) and a shoulder implant (Fig. 8) model^37^. First, in 7 mice, a dorsally placed cancellous human allograft implant was either infected with bioluminescent Xen36 (4 mice) or treated with PBS (3 mice). The 1D9-680 probe was then intravenously injected in an identical manner to that of the spine model, and all 7 mice were imaged 72 h PI (Fig. 7). As observed with the spinal model, the in vivo FLI signals corresponded closely with the in vivo BLI signal, demonstrating the 1D9-680 antibody accumulated at the infection site equally well in this animal implant model. In this case, even the weakest in vivo BLI signal (mouse #4 from the left) was accurately detected by this probe. Finally, the 1D9-680 probe was evaluated in a shoulder arthroplasty model (Fig. 8), with 4 mice infected with Xen36 and 3 mice acting as sterile controls^37^. In contrast to the spine and allograft models, where even infections with the weakest in vivo BLI signals could be detected by the 1D9-680 probe to give a concomitant in vivo FLI signal, only the strongest in vivo BLI signal could be detected above background. Whether this more limited detection is due to greater depth of tissue or less efficient labelling by the 1D9-680 probe is not known. However, tissue penetration should not pose a problem in surgical settings, where infectious sites are exposed for imaged-guided debridement of fluorescently-labelled infected tissue.

### Testing 1D9-680 for its ability to delineate infection using image-guided surgery

**Figure 9:**
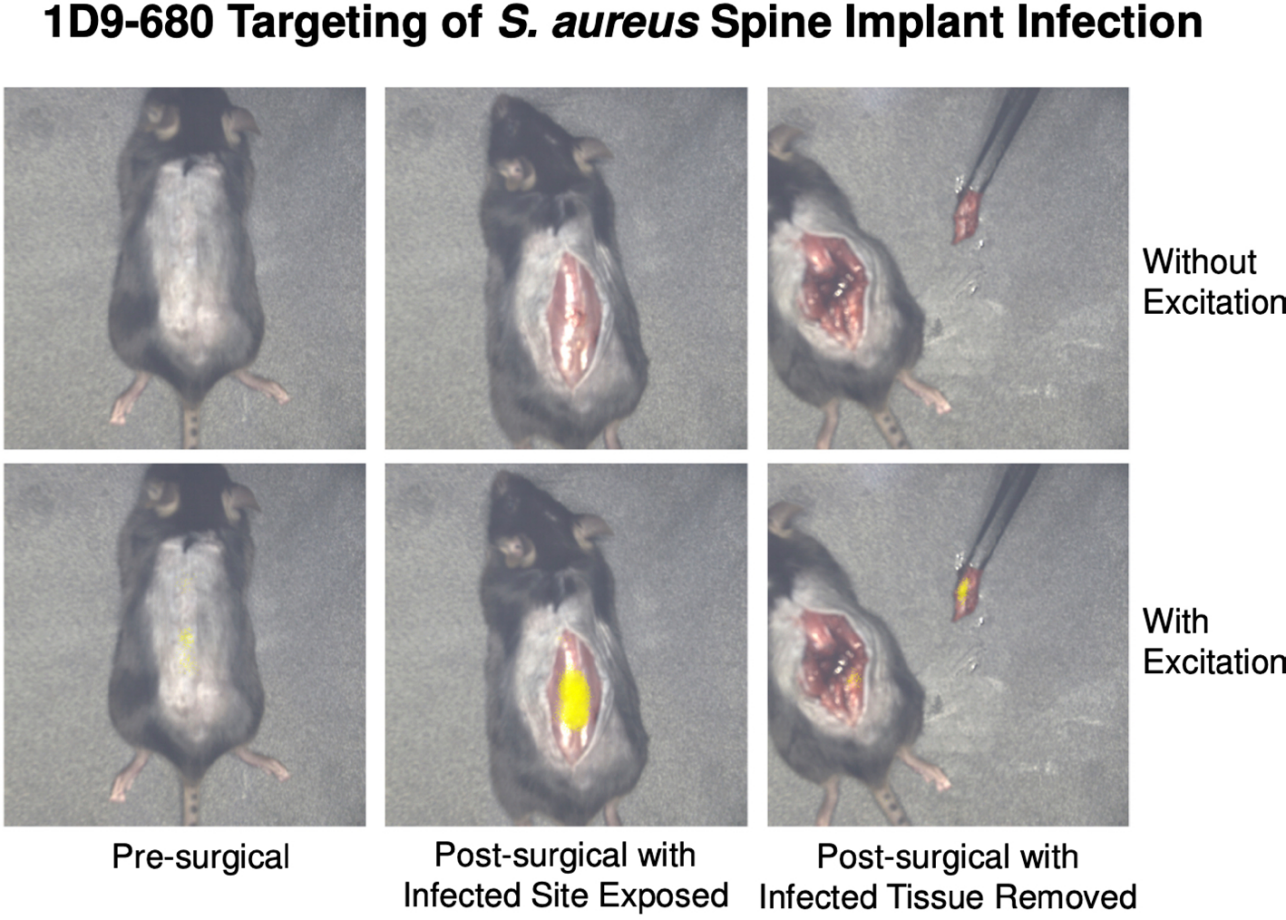
Image-guided surgical debridement of infected tissue in the spinal implant model. Image-guided surgery was performed utilizing the PerkinElmer Solaris Image-Guided Surgery System. Both the real-time white light images and fluorescence overlay images of a surgical debridement are shown. Pseudo-color yellow fluorescence can be seen in the pre-surgical panel, bottom image. Once the skin is opened, the wound bed is bright yellow in the bottom panel, which is indicative of the stronger probe signal. Following debridement of tissue, the bottom panel reveals no signal within the wound bed and persistent probe signal in the tissue specimen held by forceps.

Image-guided infection debridement surgery was performed using the Solaris Fluorescence Image-Guide Surgery System (PerkinElmer, Hopkinton, MA). This system utilizes an overhead camera with fluorescence input to display real-time images of the surgical specimen with fluorescence signal overlay. Based on the tightly correlated in vivo FLI signals of the 1D9-680 probe and in vivo BLI signals of the bacteria, image-guided surgery was performed with an excitation wavelength of 660 nm. Prior to skin incision, the in vivo FLI signals overlying the spinal surgical site was readily visualized. The skin was incised and the visualized fascia exhibited persistent in vivo FLI signals. Fluorescent tissue was sharply debrided, and the excised tissue specimen was imaged with ex vivo BLI and FLI, which yielded closely correlated signals (Fig. 9). The wound was debrided until all remaining fluorescent tissue was resected.

## Discussion

The findings presented in this study address the pressing clinical shortcomings in the diagnosis and treatment of implant infections. In cases that require surgical debridement, the basic tasks of accurate localization and identification of pathogens remain a challenge, often to the detriment of the patient^12,25^. Imaging modalities such as US, XR, CT, PET, and MRI are limited in their ability to discriminate infections from other differential processes^13–17^. Successful biopsy or culture of the bacterial foci are vital to successful treatment, yet current techniques suffer from considerable false-negative and false-positive results, which contribute to treatment failures and patient morbidity^17,18,25^. Moreover, surgical debridement is rudimentary at best, depending on the individual surgeon’s visual assessment of pathologic tissue, such as discoloration or changes in texture; this assessment is fundamentally subjective and prone to error.

In order to address these shortcomings, the purpose of this study was to compare two novel *S. aureus* targeting fluorescent imaging (TFLI) probes in their ability to identify and delineate this bacterium in implant infections, and to assess whether the more accurate probe could be utilized in a real-time, fluorescence image-guided surgical system. The two candidate probes, Vanco-800CW and 1D9-680, were both found to target *S. aureus *in vitro and in vivo with high affinity. However, the Vanco-800CW probe had background signals within the pelvis and abdomen, whereas the 1D9-680 probe specifically localized to the site of the in vivo BLI signals of the bacteria, which was most apparent upon 3D co-registration of CT and in vivo BLI/FLI analysis. The 1D9 probe demonstrated excellent specificity for *S. aureus* in three different mouse models of surgical implant infections and good sensitivity in all but the low-grade shoulder implant infection. Mice with infected spinal implants were then subjected to a fluorescence image-guided surgical system, in which the 1D9-680 probe successfully guided debridement surgery. These data provide a proof-of-concept for the specific, non-invasive diagnosis and localization of bacterial infection while leveraging TFLI within an image-guided surgery system to aid in the resection of infected tissue.

The implementation of fluorescent probes for TFLI of bacterial infections is a burgeoning theranostic approach for clinical infections. Aside from the Vanco-800CW and 1D9-680 utilized in this study, previous work has focused on probes that target the cell membrane, membrane transporters, bacterial enzymes, genetic markers, and specific organelles, in a wide range of bacteria, including *Staphylococcus, Enterococcus, Bifidobacterium, Salmonella, Mycobacterium, Klebsiella,* and *Pseudomonas* species^26–32,39^. This recent torrent of studies points to the substantial interest in targeted diagnostic and therapeutic technologies, especially with the widespread emergence of multi-drug resistant organisms that do not respond to standard antibiotics^33,34^. The development of multiple bacterial probes with unique optical outputs may enhance the ability to rapidly discriminate between bacterial pathogens and pave the way for unique therapeutic modalities. TFLI technology allows real-time, image-guided infection debridement surgery which we believe represents the next translational step of this technology. At present, no bacteria-specific molecular probes exist that are licensed for human use, although several candidates are in preclinical development. It has been postulated that the ideal probe for human use will likely be a fluorescently labelled antibody, antibiotic derivative, or modified antimicrobial peptide^34,39^. Both Vanco-800CW and 1D9-680 fit this description. However, the Vanco-800CW probe non-specifically bound to areas within the abdomen and pelvis. Based on these data, we believe 1D9-680 represents a more specific and stable probe for *S. aureus* infections, illuminating infections for at least 72 h following injection. On the other hand, it is important to bear in mind that Vanco-800CW targets a wide range of Gram-positive bacteria that cannot be detected with the IsaA target-specific 1D9-680 probe as exemplified in Fig. 1 of this study^22,23^. This view is underlined by prosthetic joint infections, which are more frequently caused by Gram-positive bacteria other than *S. aureus*^40^. In this respect, the simultaneous imaging with both the 1D9-680 and Vanco-800CW probes could offer an opportunity for in vivo diagnostics to distinguish infections caused by *S. aureus* or other Gram-positive bacteria.

There are several limitations to this animal study. First, the extrapolation of findings from animal models to human clinical conditions should be interpreted with caution. In particular, the methods implemented in this study, including the injection of probe at the known peak of infection within the animal model might not represent the most ideal conditions in which to detect bacterial infection in humans. Second, in vivo FLI is inversely related to the amount of intervening tissue, which in humans may prove to be a challenge in the diagnosis of deep-seated infections such as peri-prosthetic total hip arthroplasty infection. This issue may have been highlighted by the inability of 1D9-680 to detect a low-grade shoulder girdle infection in the mouse model, where a low bacterial burden combined with a thicker overlying tissue led to limited sensitivity.

In conclusion, the 1D9-680 probe, but not the Vanco-800CW probe, more specifically co-localized to the *S. aureus* infection in a mouse model of spinal implant infection as evidence by 2D and 3D in vivo BLI and FLI. This specificity was maintained in allograft and shoulder implant infection models. The fluorescence emitted by this probe aided in the debridement of infected tissue within an open air, fluorescence image-guided surgery system, which we believe is the next advancement in the utilization of this technology that can be translated to clinical use. These findings provide proof-of-concept of TFLI technology enhancing the diagnosis and treatment of bacterial implant infections, which are currently beleaguered by inaccurate diagnosis and empiric or incorrect treatment to the detriment of countless patients.

## Methods

### Ethics statement

All animals were maintained and utilized in accordance with good animal practice as defined in the federal regulations set forth in the Animal Welfare Act (AWA), the 1996 Guide for the Care and Use of Laboratory Animals, and PHS Policy for the Humane Care and Use of Laboratory Animals. All animal use was in accordance with institutional animal care and use committee protocols, and was approved by the UCLA Chancellor’s Animal Research Committee (ARC# 2012-104-03J).

### Bioluminescent S. aureus strain

*Staphylococcus aureus* Xen36 strain (PerkinElmer, Hopkinton, MA) is a bioluminescent derivative of the clinical isolate ATCC 49525 (Wright)^35^. *S. aureus* Xen36 possesses a Gram-positive optimized *luxABCDE* operon stably integrated into a large native plasmid^35^ yielding a maximal emission wavelength of approximately 490 nm constitutively produced by live, metabolically active bacteria. It has been previously shown to be the optimal *S. aureus* strain for use in such experiments due to the strength and consistency of its bioluminescence signal^36^. *S. aureus* Xen36 is kanamycin resistant and is grown with 200 µg/ml kanamycin (Sigma–Aldrich) to avoid contamination. *S. aureus* was streaked onto tryptic soy agar plates (tryptic soy broth [TSB] plus 1.5% bacto agar; BD Biosciences) and grown at 37 °C overnight. Single colonies of *S. aureus* were then cultured in TSB and again grown overnight at 37 °C in a shaking incubator (240 rpm) (MaxQ 4,450, Thermo Fisher Scientific). Mid-logarithmic phase bacteria were obtained after a 2 h subculture of a 1:50 dilution of the overnight culture. Bacterial cells were pelleted, re-suspended, and washed three times in PBS. Bacterial inocula of 1 × 10^3^ colony forming units (CFU) were ascertained by measuring the absorbance at 600 nm (A600, Biomate 3 [Thermo]) and comparing to a reference standard. This dose was derived from previous described methods utilizing this model^24^.

### In vitro imaging of staphylococcal biofilms

*S. aureus* Xen36 and *S. epidermidis* ATCC 38984 were grown overnight in tryptic soy broth (TSB) in a shaking incubator at 37 °C. Bacterial biofilms were grown on chemically resistant borosilicate 18 mm glass coverslips (Paul Marienfeld GmbH, Lauda-Königshofen, Germany) in a 12-well microtiter plate containing TSB supplemented with 5% glucose and 4% NaCl. To this end, the wells were inoculated from the overnight cultures to a final optical density at 600 nm (OD_600_) of 0.1, and the plates were incubated at 37 °C for 48 h. Co-cultures of *S. aureus* and *S. epidermidis* were prepared by inoculating the wells at a 1:1 ratio from the respective overnight cultures. Coverslips with biofilm were incubated with a mix of 0.2 µM 1D9-Alexa555 and 0.2 µM Vanco-BODIPY FL (Thermo Fisher Scientific) for 30 min in PBS. Subsequently, the biofilms were washed 1× with PBS to remove unbound fluorescent probes and, thereafter, they were fixed in 4% paraformaldehyde. Finally, the coverslips were mounted on microscopy slides. Image acquisition was performed with a Leica TCS SP8 confocal microscope. The recorded images were processed using ImageJ software (National Institutes of Health) and LAS X Life Science. The 1D9-Alexa555 probe used in these experiments was obtained by crosslinking the Alexa Fluor 555 dye (Thermo Fisher Scientific) to the human monoclonal antibody 1D9 via activated N-hydroxysuccinimide ester chemistry.

### Mouse model of spinal implant infection

The following description is sourced from work previously published by our group^24^. The mice used within these experiments were twelve-week-old male C57BL/6 wild-type mice (Jackson Laboratories, Bar Harbor, ME). Mice were kept at 4 per cage in standard cages with a 12 h light and dark cycle. Free access to water and standard pellet feed were utilized in compliance with the standards of the UCLA Chancellor’s Animal Research Committee. Veterinary staff ensured the well-being of all animals throughout the experiment by daily assessment. Mouse spinal implant surgeries were performed as previously described^24^. In brief, animals were shaved along the dorsum of their lumbar spine, skin was prepped with alcohol and betadine, and inhalation isoflurane (2%) was utilized for anesthesia. A dorsal skin incision ~ 2 cm in length was utilized, centered over the lower lumbar spine. The lumbar spinous processes were exposed by carefully peeling away the soft tissue from the posterior elements of the spine, unilaterally. The spinous process of L4 was reamed with a 25-gauge needle, and this reamed cavity received an “L-shaped” surgical-grade 0.1 mm diameter stainless steel implant (Modern Grinding, Port Washington, WI). The short arm of the “L-shaped” implant was placed through the reamed cavity of the spinous process, and the long arm extended toward the head of the mouse. Two 4–0 Vicryl sutures were placed in preparation for closure. An inoculation of 1 × 10^3^ CFU of bioluminescent Xen36 *S. aureus* or sterile saline was pipetted onto the bend of the implant, the previously placed sutures were expeditiously tied to avoid contamination of the wound, and a running 4–0 Vicryl suture was utilized to close the overlying skin. Buprenorphine (2.5 mg/kg) (ZooPharm, Fort Collins, CO) was administered for analgesia, subcutaneously, every 72 h for the duration of the experiment. Mice were then subjected to high resolution X ray (Faxitron LX-60 DC-12 imaging system) to confirm placement of the implant.

### Mouse model of allograft infection

As previously described, 12-week-old male C57BL/6 wild-type mice were used (Jackson Laboratories, Bar Harbor, ME) for the allograft infection experiment with a similar care protocol_._ Human fibular cortical allograft was obtained from, and sterilized in the standard manner, by Musculoskeletal Transplant Foundation (Edison, NJ). Bulk allograft was shaped into 2.5 mm diameter discs using a high-speed saw and wire cutter. Scissors were used to trim rough edges, and implants were sterilized in an autoclave. Survival surgery was performed in which the allograft was implanted in the subcutaneous space dorsal to the caudal cervical spine. Mice were anesthetized via inhalation isoflurane (2%). The level of the implantation was approximated by palpating the maximal point of lordosis with minimum skin tension. A 1 cm midline incision was then made and carried down to the fascia, exposing subcutaneous muscle. The dissection was directed bilaterally superficial to the paraspinal musculature, developing a subcutaneous pocket for the allograft implant. Fine-toothed forceps were used to gently place the sterile implant into the pocket created by dissection. An inoculation of 1 × 10^2^ CFUs of bioluminescent Xen36 *S. aureus* in 2 µL phosphate buffered solution or 2 µL sterile saline (control group) was pipetted onto the allograft. A single 4.0 Vicryl suture was then placed in a running fashion to approximate the skin. Quick-release buprenorphine (0.3 mg/kg) (Zoo-Pharm, Fort Collins, CO) was administered subcutaneously every 12 h for 72 h as post-operative analgesic.

### Mouse model of shoulder implant infection

As previously described, 12-week-old male C57BL/six wild-type mice were used (Jackson Laboratories, Bar Harbor, ME) for the shoulder implant infection experiment with a similar care protocol^37^. The mice were anesthetized for survival surgery. The shoulder joint was approximated by palpating superiorly towards the most proximal aspect of the humerus. An incision was made starting at the sternum and extending laterally across the deltoid, which exposed the deltopectoral groove beneath. Next, the pectoralis musculature was removed from its insertion on the humerus. Once near the humeral head, the joint was exposed by applying anterior directed pressure on the posterior aspect of an extended and externally rotated humerus, which subluxed the joint anteriorly. A 25-gauge needle was used to ream the most proximal aspect of the humeral head, with the needle aimed towards the palpable deltoid tuberosity. The needle was then removed, allowing the placement of the implant, with the implant’s most proximal aspect communicating with the glenoid fossa within the shoulder joint. 5–0 vicryl was then used to approximate the deep fascial layers. These sutures were placed but not tied to allow for expedient closure after inoculation and to restrict bacteria to the immediate area of the implant. Next, a 2 µL inoculation of 1 × 10^3^ (e3 group) or 1 × 10^4^ (e4 group) CFUs of bioluminescent *S*. *aureus* Xen-36 or sterile saline (control group) was pipetted onto the tip of the implant. Deep sutures were tied and a running 5–0 vicryl was used to approximate the skin. Sustained release buprenorphine (2.5 mg/kg) (Zoo-Pharm, Fort Collins, CO) was then administered subcutaneously every 72 h as analgesic for the duration of the experiment.

### Description of probes and injection protocol

The 1D9-680 probe is composed of the anti-IsaA IgG1 antibody conjugated to the near infrared fluorophore NIR680 (PerkinElmer, Hopkinton, MA) with peak excitation of 680 nm as previously described^18,23^. The IsaA epitope is a ubiquitously expressed protein involved in the cell wall metabolism of *S. aureus* and a few other staphylococcal species^23^. Vancomycin-IRDye800CW (Vanco-800CW) is a vancomycin molecule conjugated to IRDye 800CW, a near-infrared fluorophore with peak excitation at 778 nm and emission at 794 nm. Vanco-800CW was synthesized as previously described^22^. Both 1D9-680 and Vanco-800CW were administered to infected and control group mice on POD 7. To this end, the probes were dissolved in phosphate-buffered saline to deliver 25 nmol of Vanco-800CW and 0.1 μg 1D9-680 in a 100 µL final injection volume. Mice were anesthetized via inhalation isofluorane (2%) and probe solutions were administered via tail vein injection as previously described^22,23^. Mice were monitored daily for systemic reaction and adverse effects.

### Bioluminescence and fluorescence imaging

Mice were anesthetized via inhalation isoflurane (2%) and in vivo bioluminescence and fluorescence imaging was performed using an IVIS Spectrum-CT (PerkinElmer, Hopkinton, MA). Anesthesia was maintained at 1.5% during imaging. 2D Bioluminescent imaging was performed as previously described^24^. Fluorescent excitation/emission pairs of 675/720 nm and 745/800 nm were used for 1D9-680 and Vanco-800CW probes, respectively. 2D Images were obtained 24, 48, and 72 h after probe injection. 2D data are presented via color scale overlaid on a grayscale photograph of mice and quantified within a circular region of interest (16,103 pixels) as mean maximum flux (photons per second (s) per cm^2^ per steradian (sr) [p/s/cm^2^/sr]) for bioluminescence and as maximum radiant efficiency ([photons/s]/[mW/cm^2^]) for fluorescent imaging using Living Image 4.2 software (PerkinElmer, Hopkinton, MA).

At the indicated timepoints, Diffuse Light Imaging Tomography (DLIT—3D bioluminescence) images were acquired with a Spectrum-CT using emission filters that correspond to the spectral profile of *lux*. Immediately following DLIT, Fluorescence Light Imaging Tomography (FLIT—3D fluorescence) images were acquired with a Spectrum-CT using the filter pairs mentioned above over a scan field surrounding the implants. 3D images were reconstructed via tomographic algorithms and automatically coregistered with a CT scan using the Living Image software package.

### Solaris fluorescence image-guided surgery system

Image-guided operative debridement of infected tissue and implant retrieval were performed 72 h following PI utilizing a Solaris Fluorescence Image-Guided Surgery System (PerkinElmer, Hopkinton, MA) under ambient light conditions. Mice were sacrificed immediately prior to operative debridement. An excitation filter of 660 nm was utilized corresponding to the excitation wavelength of the 1D9-680 probe. Still images and videos were periodically saved throughout the procedure using the Solaris software (PerkinElmer, Hopkinton, MA).

### Statistical analysis

Data between two groups were compared by using either one or two-tailed Student’s t-test and data form three or more groups were compared using a one-way ANOVA. All data are expressed as mean ± standard error of the mean (SEM). Values of *P* < 0.05 were considered statistically significant.

## Supplementary information

Supplementary Video Legends.

Supplementary Video 1 1D9.

Supplementary Video 1 V800.

Supplementary Video 2 1D9.

Supplementary Video 2 V800.

Supplementary Information.

## Data Availability

All data generated or analyzed during this study are included in this published article (and its Supplementary Information files).
