# Supplementary material for: Comparison of two fluorescent probes in preclinical non-invasive imaging and image-guided debridement surgery of Staphylococcal biofilm implant infections
Source: Sci Rep. 2021 Jan 15;11:1622. doi: 10.1038/s41598-020-78362-7 (PMC7810895; doi:10.1038/s41598-020-78362-7)
Supplement: Supplementary file 1 — Supplementary Video Legends. [file 41598_2020_78362_MOESM1_ESM.docx]

**Supplemental Data**

**Supplemental Movie 1:**

Upon first revolution, three-dimensional CT reconstruction with bacterial bioluminescence signal emanating from the spinal implant of a representative mouse. Upon second revolution, from left to right, the same representative mouse with 1D9-680 probe and Vanco-800CW probe signal superimposed at 72 hours PI. The overlay of the 1D9-680 probe reveals co-localization while the Vanco-800CW probe reveals co-localization in addition to probe signal in various parts of the abdomen and pelvis.

**Supplemental Movie 2**

Upon first revolution, three-dimensional CT reconstruction with bacterial bioluminescence signal emanating from the spinal implant of a representative mouse, different from that of Supplemental Figure 1. Upon second revolution, from left to right, the same representative mouse (different from that of Supplemental Figure 1) with 1D9-680 probe and Vanco-800CW probe signal superimposed at 72 hours PI. The overlay of the 1D9-680 probe reveals co-localization while the Vanco-800CW probe reveals co-localization in addition to probe signal in various parts of the abdomen and pelvis.
