# Supplementary figures and images for: Comparison of two fluorescent probes in preclinical non-invasive imaging and image-guided debridement surgery of Staphylococcal biofilm implant infections
Source: Sci Rep. 2021 Jan 15;11:1622. doi: 10.1038/s41598-020-78362-7 (PMC7810895; doi:10.1038/s41598-020-78362-7)

## Slide 1
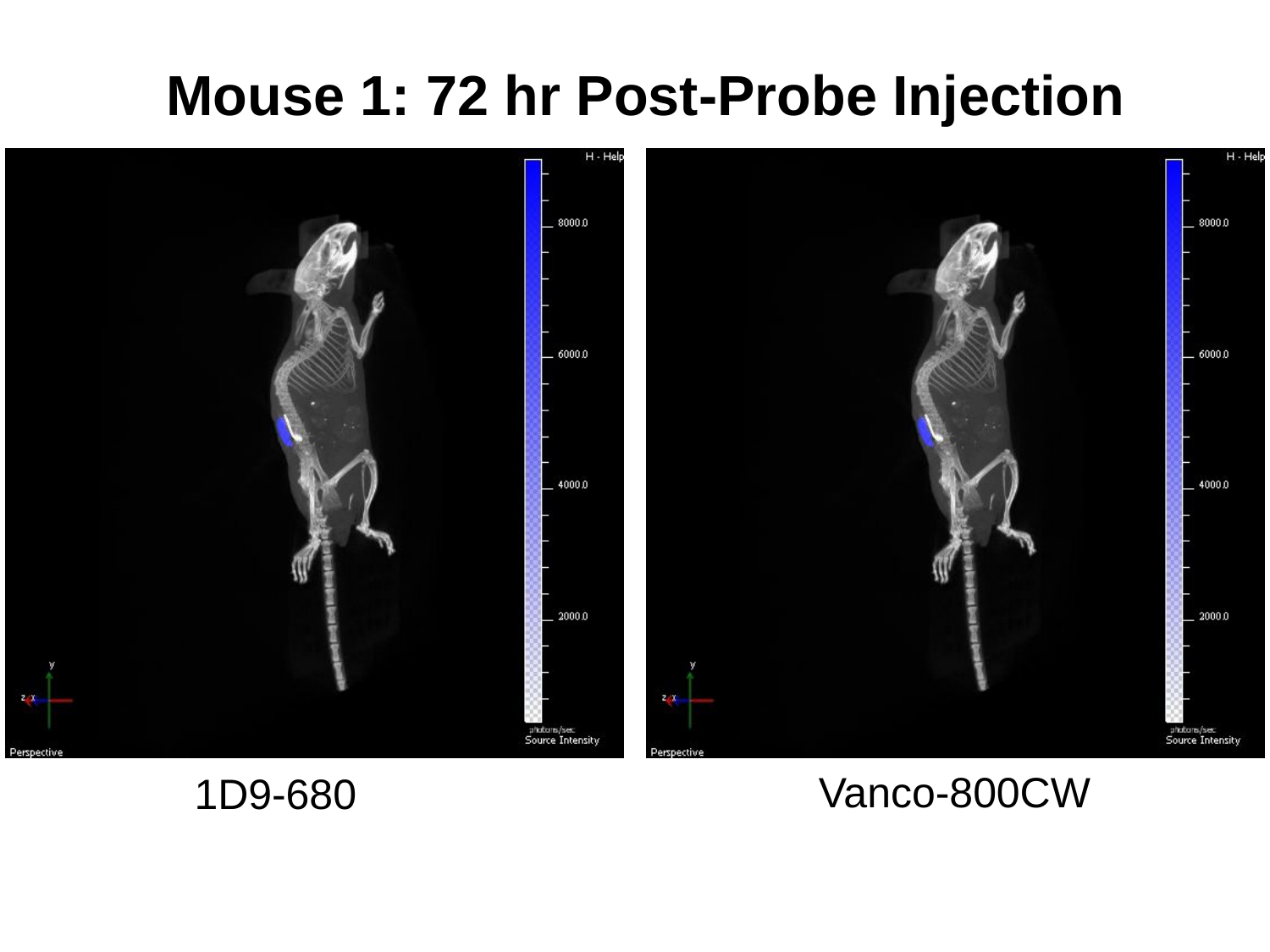

Mouse 1: 72 hr Post-Probe Injection
Vanco-800CW
1D9-680

## Slide 2
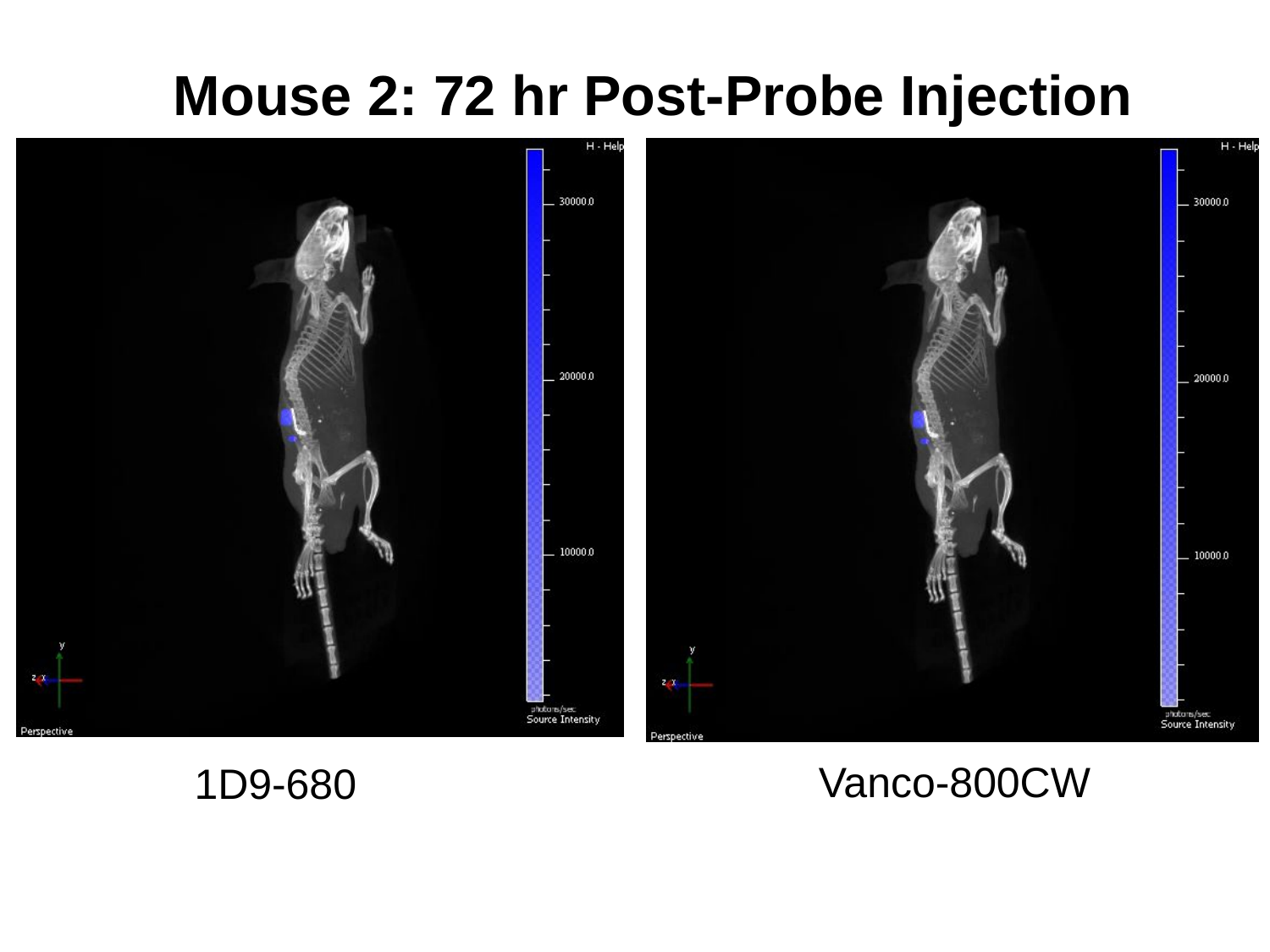

Mouse 2: 72 hr Post-Probe Injection
Vanco-800CW
1D9-680

Supplement: Supplementary file 6 — Supplementary Information. [file 41598_2020_78362_MOESM6_ESM.pptx]
